# OLINK proteomics identifies inflammatory protein signatures associated with vascular cognitive impairment in diabetes

**DOI:** 10.3389/fimmu.2026.1677578

**Published:** 2026-05-22

**Authors:** Yuying Wang, Zhenyu Zhao, Linna Ji, Haoying He, Sisi Peng, Juan Xu, Zhipeng Xu, Junjian Zhang

**Affiliations:** 1 Department of Neurology, Zhongnan Hospital of Wuhan University, Wuhan, China; 2 Hubei Provincial Clinical Research Center for Dementia and Cognitive Impairment, Wuhan, China

**Keywords:** biomarker, diabetes mellitus, inflammation, proteomics, vascular cognitive impairment

## Abstract

**Introduction:**

Vascular cognitive impairment (VCI) is a syndrome of cognitive dysfunction attributable to vascular risk factors and cerebrovascular diseases, representing a major component of global dementia burden. Dysglycemia, encompassing diabetes mellitus and impaired glucose regulation, is increasingly recognized as a modifiable risk factor for cognitive decline, particularly VCI. Chronic low-grade inflammation mediates the association between metabolic dysfunction and neurodegeneration. The lack of validated diagnostic tools for early VCI detection in high-risk dysglycemic populations highlights the urgent need for robust diagnostic models based on molecular signatures.

**Methods:**

We analyzed serum samples from 94 participants, categorized into three groups: normal glucose with normal cognition (NG-NC), dysglycemia with normal cognition (Dys-NC), and dysglycemia with VCI (Dys-VCI). Using the Olink Target 96 Inflammation Panel, we quantified 92 inflammation-related proteins. Differentially expressed proteins (DEPs) were identified as potential biomarkers, followed by functional enrichment analysis to explore associated biological pathways. Logistic regression models, combined with ROC analysis, assessed the diagnostic utility of selected protein panels across groups.

**Results:**

Compared with NG-NC, the Dys-NC group exhibited upregulated pro-inflammatory mediators (CXCL1, CXCL5, OSM) and downregulated anti-inflammatory proteins (FGF-21, AXIN1). The Dys-VCI group showed significant increases in TNFB, IL-12B, TNF, and CSF-1. Key proteins, including AXIN1 and CX3CL1, displayed progressive changes across the metabolic-cognitive spectrum. Pathway analysis revealed enrichment in cytokine-cytokine receptor interaction, viral protein interaction with cytokine and cytokine receptor, TNF signaling, and chemokine signaling pathways. A four-protein panel (CCL3, CX3CL1, FGF-21, CXCL1) achieved an area under the curve (AUC) of 0.903 for distinguishing Dys-NC from NG-NC, while another panel (TNFB, IL10, IL-12B, CX3CL1) demonstrated an AUC of 0.799 for identifying Dys-VCI among Dys-NC.

**Discussion:**

Our study identified unique inflammatory protein profiles associated with different metabolic and cognitive states, providing insights into inflammatory mechanisms linking dysglycemia and VCI, and highlighting potential biomarker candidates for longitudinal validation.

## Graphical abstract

**Figure:**
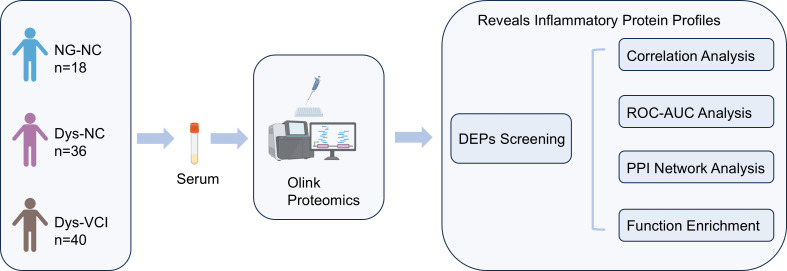

## Introduction

Globally, approximately 50 million people worldwide are affected by dementia, and this number is projected to triple by 2050 (1). Vascular cognitive impairment (VCI) is the second most common cause of dementia following Alzheimer’s disease (AD), accounting for at least 20-40% of all dementia cases (2). VCI represents a heterogeneous clinical syndrome that includes all aspects of cognitive dysfunction from mild cognitive impairment to vascular dementia (3). The landmark Leukoaraiosis and Disability (LADIS) study established white matter changes as independent predictors of cognitive decline and dementia progression, highlighting the clinical significance of vascular brain injury in aging populations (4, 5).

VCI is caused by ischemic or hemorrhagic cerebrovascular lesions, either acting alone or in combination with neurodegenerative processes, including those associated with AD (6). Established risk factors for VCI include advanced age, hypertension, diabetes mellitus, hyperlipidemia, atrial fibrillation, smoking, and prior cerebrovascular events (7). These factors often interact synergistically, with multiple comorbidities substantially amplifying the risk of cognitive decline. Chronic cerebral hypoperfusion plays a significant role in VCI, triggering neuroinflammation, oxidative stress, and blood-brain barrier disruption, thereby leading to neuronal damage and worsening cognitive dysfunction (8). Current VCI biomarker research spans multiple molecular pathways, including neurodegeneration markers such as neurofilament light chain (NfL) and glial fibrillary acidic protein (GFAP), endothelial dysfunction indicators, inflammatory cytokines, matrix metalloproteinases (MMPs), metabolites like homocysteine, and microRNAs (miRNAs) (2, 9–11). However, single biomarkers lack specificity due to VCI’s multifactorial nature, driving research toward multi-pathway biomarker panels and machine learning approaches to enhance diagnostic accuracy.

Diabetes mellitus (DM) is characterized by chronic hyperglycemia and a rising prevalence, making it a major global public health issue. China bears the largest diabetic burden worldwide, with over 118 million cases, accounting for approximately 22% of the global total (12). Diabetes confers a significantly elevated risk for vascular cognitive impairment, with a 2.27-fold increased risk for vascular dementia and a 1.53-fold higher likelihood of progression from mild cognitive impairment to dementia, highlighting the critical intersection of metabolic dysregulation and cerebrovascular pathology (13). In this study, dysglycemia is defined as abnormal glucose metabolism encompassing both diabetes mellitus and prediabetes states, based on the American Diabetes Association (ADA) diagnostic criteria (14). Long-term dysglycemia has been increasingly recognized as a risk factor for accelerated cognitive decline (15, 16). Furthermore, cognitive dysfunction emerged as a key complication associated with DM, significantly impairing quality of life and imposing a heavy socioeconomic burden (17). Mounting evidence suggests that dysglycemia, encompassing conditions such as DM, impaired fasting glucose (IFG), impaired glucose tolerance, and metabolic syndrome, is strongly associated with accelerated cognitive decline and represents a significant modifiable risk factor for dementia, particularly VCI (18, 19). The underlying mechanisms connecting diabetes and neurodegenerative disorders involve insulin resistance, endothelial dysfunction, and notably, chronic low-grade inflammation (20, 21). Systemic inflammation has been proposed as a potential mechanistic link between diabetes and neurodegenerative disorders (22, 23). Despite accumulating evidence linking neuroinflammation in both DM and VCI, the precise molecular mechanisms and critical biomarkers remain incompletely understood.

The development of accurate diagnostic models for VCI in dysglycemic patients represents a critical unmet clinical need. Current diagnostic approaches rely heavily on cognitive assessments and neuroimaging, which often detect changes only at advanced stages when therapeutic interventions have limited efficacy (24). Therefore, identifying molecular signatures that enable early diagnosis through accessible biofluid-based tests could transform clinical management of at-risk populations. Recent advances in high-throughput proteomics have advanced our ability to uncover disease pathogenesis and identify novel biomarkers. The Olink platform integrates proximity extension assay (PEA) with high-sensitivity protein detection, enabling precise profiling of systemic inflammatory pathways. In this study, we utilized Olink proteomics to investigate circulating inflammatory protein signatures across distinct clinical stages spanning from normal glucose metabolism and cognition to dysglycemia and associated cognitive impairment. By employing a multi-omics approach, we aimed to identify biological pathways linked to differentially expressed proteins and uncover potential serum biomarkers for elucidating inflammatory mechanisms underlying the progression from metabolic disturbance to cognitive decline.

## Materials and methods

### Ethics approval and consent to participate

This study was approved by the Medical Ethics Committee of Zhongnan Hospital of Wuhan University (No. 2022172) prior to data collection. Informed consent forms were obtained from all enrolled patients.

### Participants

We collected serum samples from 18 healthy subjects and 76 patients with diabetes or impaired fasting glucose, including 36 patients with normal cognition and 40 patients with vascular cognitive impairment (VCI). The participants were categorized into three groups: glucose-normal controls with normal cognition (NG-NC), dysglycemia with normal cognition (Dys-NC), and dysglycemia with VCI (Dys-VCI). Serum samples from each group were analyzed using the Olink Proteomics.

Inclusion criteria were as follows: 1) 50–75 years old. 2) Ability to provide informed consent and willingness to participate in all required study procedures, including laboratory tests, neuropsychological assessments, and neuroimaging evaluations. 3) No history of severe systemic diseases, and capable of completing standardized laboratory tests, neuropsychological assessments, and neuroimaging evaluations. 4) Participants in the dysglycemia group met diagnostic criteria for either DM or prediabetes. Specifically, DM was diagnosed as fasting plasma glucose (FPG) ≥7.0 mmol/L or HbA1c ≥6.5%, while prediabetes was defined as FPG between 5.6–6.9 mmol/L or HbA1c between 5.7% and 6.4%, according to the American Diabetes Association (ADA) diagnostic criteria (14). 5) VCI was diagnosed according to the Chinese Expert Consensus on the Diagnosis and Management of Vascular Cognitive Impairment (2024 Edition) (25). Cognitive function was assessed using the Montreal Cognitive Assessment (MoCA). Cognitive impairment was defined based on education level: university/college: MoCA score ≤25; professional qualification/secondary school: MoCA score ≤24; primary school: MoCA score ≤23 (26, 27). Neuroimaging confirmation was performed using 3T brain MRI, where white matter hyperintensities were rated using the Fazekas scale (score ≥2 considered significant) and the presence of lacunar infarcts was documented. Final diagnosis was confirmed by an experienced neurologist based on clinical, neuropsychological, and neuroimaging findings. Exclusion criteria were as follows: 1) Presence of severe psychiatric disorders or neurodegenerative diseases (e.g., Alzheimer’s disease, Parkinson’s disease). 2) Severe psychiatric disorders (e.g., major depressive disorder, anxiety disorder), determined by HAMD and HAMA scores. 3) Acute systemic infection or current use of immunosuppressive therapy. 4)Severe hepatic or renal insufficiency (e.g., cirrhosis, end-stage renal disease) or other major organ complications. 5) Pregnancy or lactation. 6) History of stroke or TIA within the past 6 months. 7) Use of anti-inflammatory drugs, corticosteroids, statins, or psychotropic medications.

### Serum sample collection

We collected 5 mL of peripheral venous blood from healthy subjects and patients with diabetes or impaired fasting glucose into EDTA tubes. We then centrifuged the samples at 3,000 × g for 15 minutes to isolate the serum, which we stored at –80 °C for later stored at -80 °C until further analysis.

### Analysis of inflammation-related proteins

The Olink Target 96 Inflammation panel (Olink Proteomics, LC-Bio Technology Co., Ltd., Hangzhou, China) was used to quantify protein levels according to the manufacturer’s instructions. The final measurements were expressed as normalized protein expression values in log2 scale. Each protein assay included in the panel underwent rigorous validation for analytical performance, including assessment of specificity, sensitivity, dynamic range, precision, scalability, endogenous interference, and detectability (http://www.olink.com). Differentially expressed proteins (DEPs) in the Olink dataset were identified using t-tests, with statistical significance set at P < 0.05. Volcano plots were generated using GraphPad Prism 9. Visualization tools, including heatmaps and Partial Least Squares Discriminant Analysis (PLSDA), were created using the ggplot2 package in R. Functional annotation and pathway enrichment analysis were performed using Gene Ontology (GO) and Kyoto Encyclopedia of Genes and Genomes (KEGG) databases to identify biological processes and pathways associated with the identified proteins. Correlation analysis was conducted to evaluate the relationship between the expression levels of any two proteins. To assess diagnostic performance, receiver operating characteristic (ROC) curves were calculated using the SPSS software package. The optimal diagnostic efficiency was visualized by plotting ROC curves using GraphPad Prism 9.

### Statistical analysis

Statistical analyses were performed using SPSS (SPSS Inc., Chicago, IL) and R software (version 4.2), with a significance level set at P < 0.05. Continuous variables were expressed as mean ± standard deviation (SD) and compared using t-tests or ANOVA if normally distributed; otherwise, the Mann-Whitney U test was applied. Categorical data were analyzed using the chi-square test. Differentially expressed proteins (DEPs) between target groups were first identified using stringent statistical criteria (p<0.05). Binary logistic regression analyses were then performed for each candidate protein to determine those with significant discriminatory power (Wald test p<0.05), followed by ROC curve analysis. The area under the ROC curve (AUC) was used to assess diagnostic performance, ranging from 0% to 100%, with higher values indicating better discriminatory ability. The optimal cutoff was selected based on the maximum Youden index (sensitivity + specificity − 1).

## Results

### Characteristics of the study subjects

A total of 18 healthy controls (NG-NC group) and 76 dysglycemia patients (including DM and IFG) were enrolled in this study. Among the dysglycemia group, 36 individuals had normal cognitive function (Dys-NC group) and 40 were diagnosed with VCI (Dys-VCI group). The clinical characteristics and peripheral venous blood sample information for all three groups were summarized in **Table 1**. There were no significant differences among the groups in age, sex, body mass index (BMI), education level, smoking or alcohol history, blood pressure, lipid profiles, or scores on the Hamilton Anxiety Rating Scale (HAMA) and Hamilton Depression Rating Scale (HAMD). However, meaningful differences were observed in fasting blood glucose (FPG), HbA1c levels, and cognitive scores as measured by the Montreal Cognitive Assessment (MoCA).

**Table 1:** Comparison of clinical data among three verified group.

Characteristics	NG-NC (n=18)	Dys-NC (n=36)	Dys-VCI (n=40)	P value
Age (years)	67.1±5.4	65.2±6.7	65.2±5.3	0.466
Sex (males, %)	55.6	50.0	57.5	0.801
BMI (kg/m^2^)	25.1±3.2	25.1±3.0	25.6±4.6	0.791
Education (%)				0.126
Primary school	11.10	0.00	5.00	
Secondary school	16.70	22.20	32.50	
Professional qualification	55.60	36.10	27.50	
University/college	16.70	41.70	35.00	
Smoking status (%)				0.430
Non-smoker	55.6	61.1	42.5	
Smoker	22.2	19.4	20.0	
Ex-smoker	22.2	19.4	37.5	
Drinker (%)				0.839
Non-drinker	61.1	72.2	70.2	
Drinker	22.2	19.4	18.1	
Ex-drinker	16.7	8.3	11.7	
Hypertension (%)	61.1	75.0	60.0	0.344
SBP (mmHg)	134.1±14.4	138.2±12.3	139.2±14.4	0.412
DBP (mmHg)	84.4±12.2	86.4±9.8	81.0±11.4	0.095
Hyperlipidemia (%)	33.3	52.8	37.5	0.275
TC (mmol/l)	4.5(3.9, 5.0)	4.5 (4.0, 5.4)	4.0(3.5, 5.1)	0.168
TG (mmol/l)	1.3(0.9, 1.7)	1.6(1.1, 2.0)	1.3(1.0, 2.0)	0.258
LDL (mmol/l)	2.3(1.8, 3.0)	2.6(2.0, 3.2)	2.4(2.1, 3.2)	0.724
HDL (mmol/l)	1.2(1.1, 1.5)	1.3(1.0, 1.5)	1.1(0.9, 1.3)	0.011
FBG (mmol/l)	4.8±0.4	6.3±1.3	7.1±1.6	<0.001
HbA1c (%)	5.4±0.1	6.3±0.8	7.3±1.5	<0.001
MoCA	26.2±1.3	26.0±1.6	17.7±4.5	<0.001
HAMA	4.0(2.0, 9.0)	6.0(1.0, 9.0)	3.0(2.0, 7.0)	0.967
HAMD	2.0(1.0, 5.0)	3.0(1.0, 7.0)	2.0(0.0, 5.0)	0.998

NG-NC, normal glucose with normal cognitive function group; Dys-NC, dysglycemia with normal cognitive function group; Dys-VCI, dysglycemia with vascular cognitive impairment group. Data are means ± SD, median (quartile range), or n (%).

BMI, body mass index; SBP, systolic blood pressure; DBP, diastolic blood pressure; TC, total cholesterol; TG, triglycerides; LDL, low-density lipoprotein; HDL, high-density lipoprotein; FBG, fasting blood glucose; HbA1c, glycated hemoglobin; MoCA, Montreal Cognitive Assessment. HAMA, Hamilton Anxiety Rating Scale; HAMD, Hamilton Depression Rating Scale.

**Figure 1:**
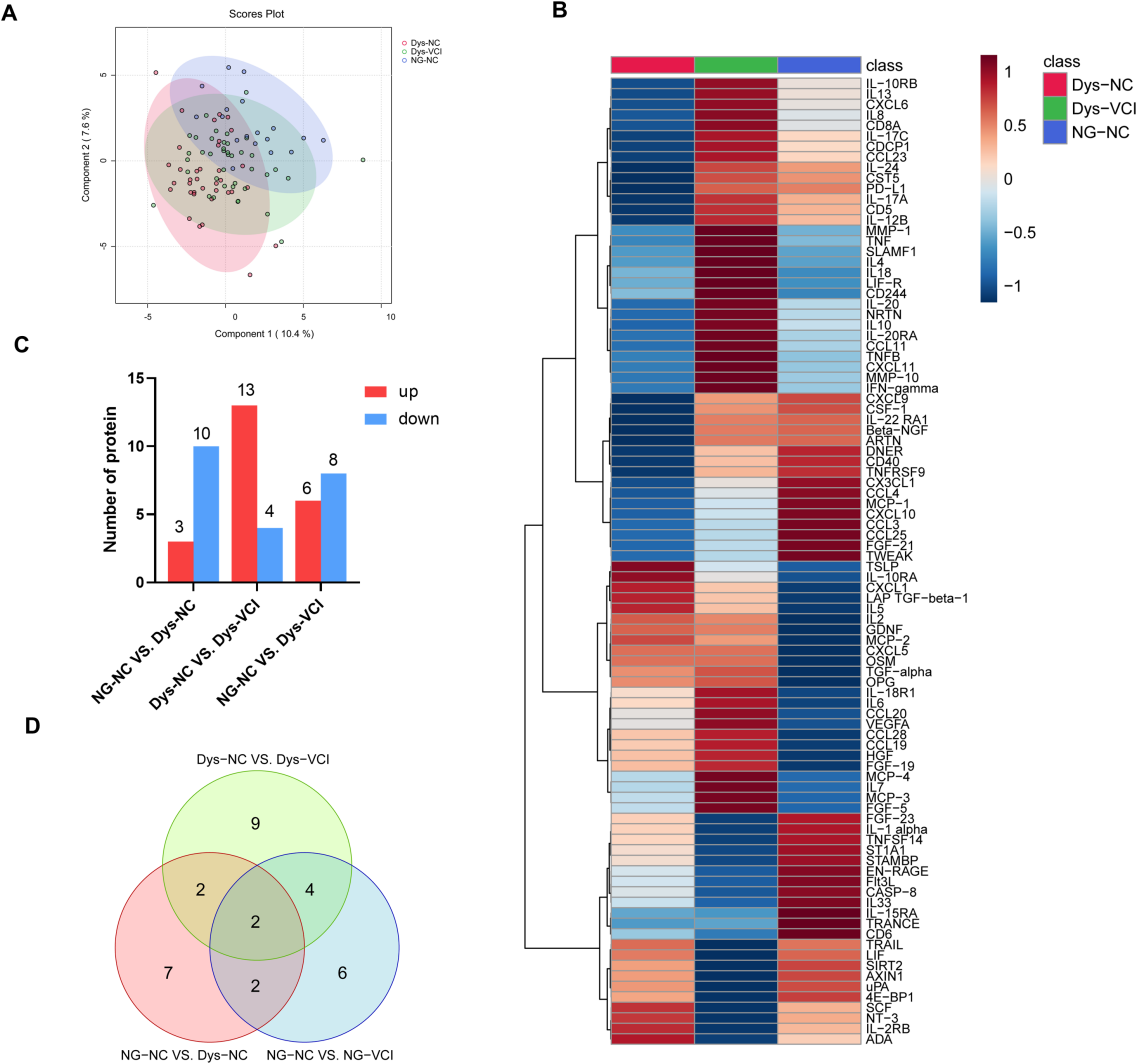
Comparison of the inflammation-related proteins among three verified groups. **(A)** Partial Least Squares Discriminant Analysis (PLS-DA) score plot showing separation of three groups along two principal components. Each dot represents an individual subject, with similar protein expression profiles clustering closely together. **(B)** Heatmap of protein expression levels across the three groups. The x-axis indicates sample groups, and the y-axis lists differentially expressed proteins. Color intensity from blue to red reflects low to high protein expression. **(C)** Comparison of numbers of differentially expressed proteins between paired groups. The bars represent proteins upregulated (red) or downregulated (blue) in the second-named group relative to the first-named group in each comparison pair. **(D)** Venn diagram illustrating the overlap in differentially expressed proteins among paired comparisons. (NG-NC, normal glucose with normal cognitive function group; Dys-NC, dysglycemia with normal cognitive function group; Dys-VCI, dysglycemia with vascular cognitive impairment group.).

Partial Least Squares Discriminant Analysis (PLS-DA) was used to examine similarities among the three groups. The first component explained 10.4% of the variance, and the second component explained 7.6% (**Figure 1A**). **Figure 1B** presents the protein expression profiles across the three groups as a heatmap. Additionally, we identified the number of upregulated and downregulated proteins in pairwise comparisons (**Figure 1C**) and used Venn diagrams to illustrate the shared differentially expressed proteins between two or three groups (**Figure 1D**).

### Comparison between the NG-NC and Dys-NC groups

**Figure 2:**
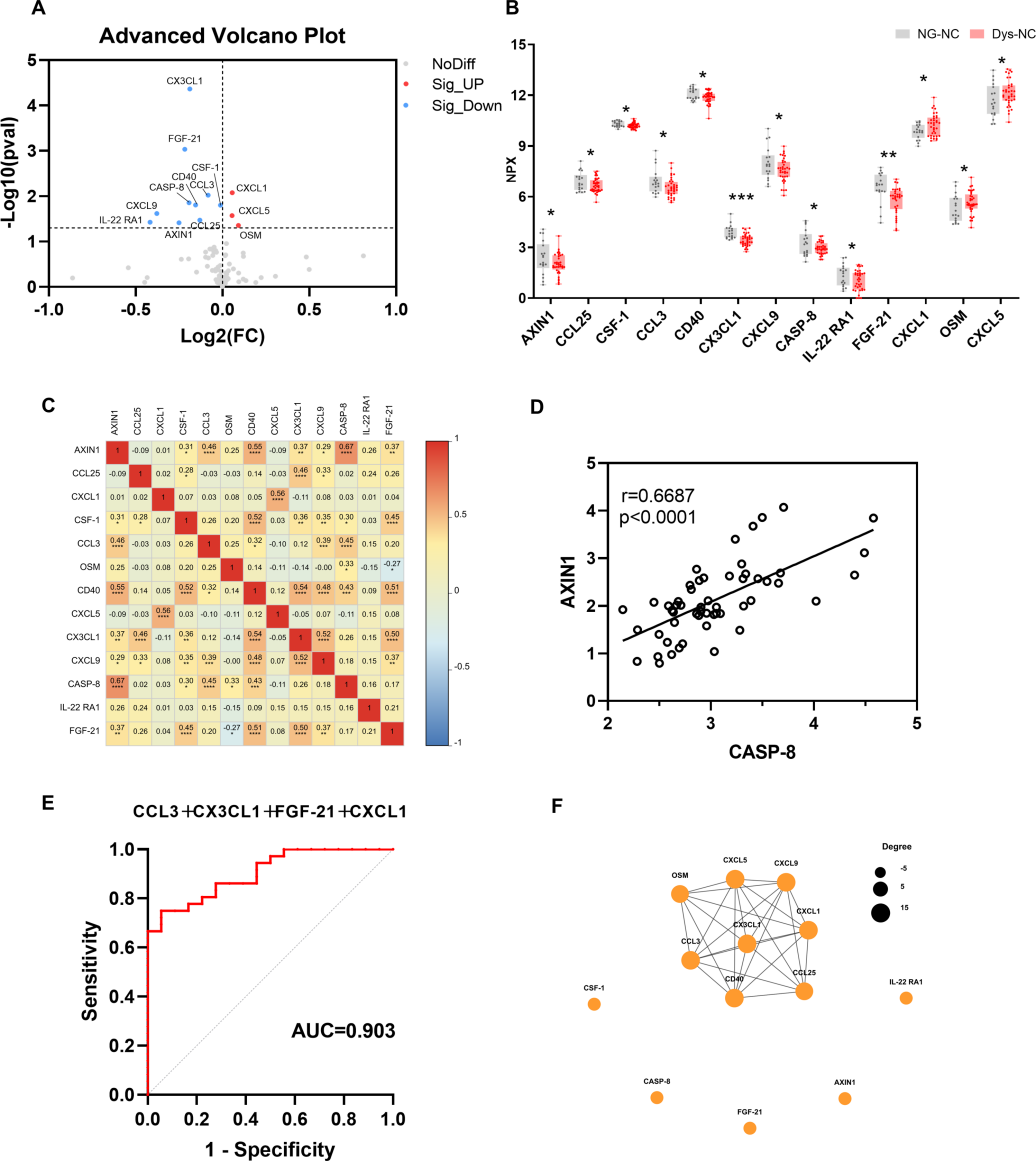
Comparison of the inflammation-related proteins between NG-NC and Dys-NC groups. **(A)** Volcano plot illustrating the differential expression of cytokines between the Dys-NC and NG-NC groups analyzed by unpaired Student’s t-test. Proteins significantly upregulated in the Dys-NC group relative to NG-NC are highlighted in red, whereas those downregulated are shown in blue. **(B)** Box plot representing differentially expressed proteins in the Dys-NC group compared to the NG-NC group with data presented as mean ± standard deviation (SD) and statistical comparisons performed using unpaired Student’s t-test. **(C)** Heatmap visualizing correlation analysis among DEPs calculated using Pearson correlation coefficients. The color scale indicates the strength and direction of correlations, with red indicating positive correlations and blue indicating negative correlations. **(D)** Scatter plot depicting the correlation between CASP-8 and AXIN1 expression levels in the study population with Pearson correlation coefficient r and P value reported. **(E)** ROC curve showing the diagnostic performance of the four-protein panel (CCL3, CX3CL1, FGF-21, and CXCL1) in distinguishing Dys-NC from NG-NC. **(F)** The PPI network between all the DEPs was established by using the STRING database. (NG-NC, normal glucose with normal cognitive function group; Dys-NC, dysglycemia with normal cognitive function group. *<0.05; **<0.01; ***<0.001).

We used the Olink platform to compare the expression levels of inflammation-related proteins between the NG-NC and Dys-NC groups (**Table 2**). As shown in **Table 3**, we identified thirteen differentially expressed proteins (DEPs) in the Dys-NC group compared with the NG-NC group: ten were downregulated (AXIN1, CCL25, CSF-1, CCL3, CD40, CX3CL1, CXCL9, CASP-8, IL-22RA1, FGF-21), and three were upregulated (CXCL1, OSM, CXCL5) (**Figures 2A, B**). We performed correlation analysis among these DEPs and visualized the results using a heatmap (**Figure 2C**). The strongest correlation was observed between CASP-8 and AXIN1 (r = 0.67, p < 0.0001) (**Figure 2D**). Using binary logistic regression, we identified four DEPs with potential diagnostic value: CCL3, CX3CL1, FGF-21, and CXCL1. ROC curve analysis demonstrated their diagnostic performance, with AUC values, 95% confidence intervals, and p-values summarized in **Table 4**. The optimal cutoff NPX values based on the Youden index were as follows: 4.659 for CCL3, 1.741 for CX3CL1, 1.648 for FGF-21, and 10.477 for CXCL1 (**Table 4**). Notably, the combination of these four DEPs showed the highest diagnostic accuracy, with an AUC of 0.903 (95% CI, 0.826- 0.980, p < 0.001) (**Table 4**, **Figure 2E**). Furthermore, to explore potential interactions among the DEPs, we constructed a protein-protein interaction (PPI) network. The network displayed a highly interconnected core module where CXCL5, CXCL9, CD40, and CX3CL1 served as central hub proteins with the highest connectivity, collectively forming the structural and functional core of the network (**Figure 2F**).

**Table 2:** Protein expression analysis among three groups.

Protein	Groups			P value		
	NG-NC (n=18)	Dys-NC (n=36)	Dys-VCI (n=40)	NG-NC VS. Dys-NC	Dys-NC VS. Dys-VCI	NG-NC VS. Dys-VCI
OPG	10.22	10.32	10.34	0.20	0.84	0.23
CXCL11	8.68	8.60	8.84	0.76	0.16	0.50
TRANCE	5.03	4.90	4.89	0.44	0.98	0.39
AXIN1	2.38	2.00	1.65	0.04	0.01	0.00
CCL25	6.90	6.66	6.74	0.03	0.45	0.26
TWEAK	10.21	10.15	10.17	0.45	0.77	0.52
TNFSF14	6.11	6.04	5.97	0.72	0.69	0.56
STAMBP	5.79	5.50	5.20	0.15	0.01	0.00
FGF-19	7.38	7.56	7.63	0.44	0.71	0.28
IL33	-0.26	-0.30	-0.30	0.62	0.99	0.64
uPA	9.28	9.28	9.25	0.98	0.72	0.73
ADA	4.02	4.07	3.93	0.58	0.10	0.36
TGF-alpha	3.68	3.77	3.79	0.66	0.88	0.60
TGF-beta1	6.55	6.64	6.61	0.49	0.79	0.62
Beta-NGF	0.39	0.39	0.39	0.80	0.97	0.77
TNFB	5.08	5.04	5.22	0.69	0.01	0.15
TNF	5.20	5.18	5.34	0.80	0.02	0.22
IFNgamma	7.42	7.34	7.68	0.80	0.17	0.39
IL-1 alpha	0.67	0.94	0.81	0.22	0.48	0.49
CD8A	8.38	8.27	8.51	0.57	0.13	0.51
CXCL10	2.50	2.33	2.39	0.22	0.57	0.38
MMP-1	16.44	16.44	16.48	0.98	0.83	0.82
IL4	-0.44	-0.33	-0.28	0.62	0.81	0.38
IL5	0.52	0.92	1.19	0.21	0.48	0.18
IL6	2.71	2.89	3.07	0.49	0.44	0.21
CD5	5.88	5.78	5.90	0.36	0.15	0.87
MMP-10	9.75	9.72	9.86	0.85	0.30	0.51
CXCL1	9.85	10.24	10.12	0.01	0.46	0.16
CSF-1	10.30	10.21	10.29	0.02	0.04	0.82
IL8	7.97	7.82	8.13	0.42	0.02	0.48
CCL3	6.92	6.54	6.67	0.01	0.37	0.22
FGF-5	1.71	1.74	1.80	0.70	0.33	0.23
IL7	2.51	2.54	2.62	0.83	0.40	0.39
CCL4	8.25	7.97	8.09	0.17	0.39	0.37
MCP-1	12.47	12.36	12.40	0.34	0.62	0.51
OSM	5.30	5.65	5.69	0.04	0.83	0.13
HGF	9.16	9.30	9.37	0.11	0.38	0.04
IL-2RB	0.14	0.08	0.04	0.63	0.74	0.42
LIF	-0.90	-0.96	-1.05	0.77	0.33	0.39
VEGFA	11.04	11.11	11.18	0.70	0.59	0.43
NT-3	3.59	3.66	3.47	0.63	0.12	0.33
SCF	9.66	9.68	9.57	0.79	0.16	0.40
IL10	4.35	4.17	4.72	0.15	0.00	0.25
CD40	12.08	11.87	12.02	0.02	0.13	0.64
CST5	6.22	6.01	6.25	0.13	0.04	0.86
IL-12B	6.30	6.06	6.39	0.20	0.01	0.65
CD6	5.38	5.33	5.32	0.64	0.91	0.59
IL13	0.29	0.34	0.39	0.80	0.69	0.55
GDNF	1.97	2.14	2.10	0.21	0.63	0.20
LIF-R	3.26	3.28	3.40	0.81	0.01	0.03
CXCL5	11.67	12.13	12.13	0.03	0.99	0.02
Flt3L	9.46	9.35	9.28	0.34	0.41	0.10
ST1A1	7.99	7.55	7.14	0.23	0.19	0.01
TRAIL	8.93	8.93	8.86	0.97	0.26	0.30
CCL11	7.64	7.61	7.70	0.79	0.23	0.52
CCL23	11.16	11.10	11.20	0.56	0.23	0.65
IL2	0.26	0.33	0.33	0.58	0.98	0.58
CX3CL1	3.91	3.43	3.67	0.00	0.00	0.02
CCL20	7.05	7.37	7.67	0.40	0.30	0.04
MCP-2	9.35	9.61	9.56	0.31	0.83	0.40
MCP-3	2.68	2.67	2.76	0.96	0.39	0.61
CXCL6	8.26	8.17	8.36	0.60	0.19	0.60
EN-RAGE	4.66	4.47	4.44	0.43	0.85	0.44
TNFRSF9	6.79	6.61	6.75	0.16	0.24	0.81
CXCL9	7.98	7.56	7.90	0.02	0.02	0.72
IL-10RB	6.94	6.91	6.95	0.83	0.64	0.89
IL-24	-0.01	-0.03	0.03	0.90	0.58	0.77
IL-15RA	1.60	1.49	1.52	0.17	0.72	0.50
SLAMF1	2.11	2.09	2.18	0.82	0.29	0.59
IL-18R1	9.11	9.23	9.32	0.20	0.25	0.02
4E-BP1	5.92	5.78	5.27	0.69	0.01	0.00
IL-10RA	0.96	0.66	0.45	0.38	0.29	0.01
IL18	9.10	9.12	9.29	0.83	0.09	0.09
CASP-8	3.25	2.95	2.78	0.01	0.11	0.00
IL-17A	1.69	1.52	1.79	0.47	0.16	0.67
ARTN	0.84	0.71	0.85	0.25	0.16	0.90
SIRT2	2.96	2.59	2.07	0.24	0.01	0.00
IL-22 RA1	1.34	1.00	1.30	0.04	0.05	0.86
DNER	7.99	7.97	7.99	0.74	0.77	0.92
TSLP	0.82	0.90	0.71	0.72	0.27	0.61
MCP-4	15.92	15.99	16.13	0.69	0.31	0.19
CCL19	12.32	12.53	12.61	0.52	0.76	0.27
NRTN	-0.59	-0.64	-0.40	0.67	0.10	0.34
CD244	6.05	6.07	6.18	0.82	0.24	0.15
FGF-23	1.92	1.88	1.87	0.71	0.94	0.74
CDCP1	5.30	5.16	5.40	0.41	0.03	0.51
CCL28	2.10	2.28	2.37	0.18	0.40	0.01
FGF-21	6.53	5.62	5.99	0.00	0.18	0.13
IL-20	-0.28	-0.20	-0.33	0.79	0.52	0.58
PD-L1	6.29	6.14	6.31	0.17	0.04	0.90
IL-17C	3.23	3.04	3.40	0.35	0.02	0.46
IL-20RA	0.37	0.52	0.40	0.41	0.34	0.74

NG-NC, normal glucose with normal cognitive function group; Dys-NC, dysglycemia with normal cognitive function group; Dys-VCI, dysglycemia with vascular cognitive impairment group.

**Table 3:** Significantly changed serum inflammatory proteins between the NG-NC group and the Dys-NC group.

Protein symbol	Uniprot ID	Name	Log_2_ FC	P value
AXIN1	O15169	Axin-1	-0.251	0.048
CCL25	O15444	C-C motif chemokine 25	-0.050	0.044
CSF-1	P09603	Macrophage colony-stimulating factor 1	-0.012	0.046
CCL3	P10147	C-C motif chemokine 3	-0.083	0.035
CD40	P25942	Tumor necrosis factor receptor superfamily member 5	-0.025	0.046
CX3CL1	P78423	Fractalkine	-0.188	0.000
CXCL9	Q07325	C-X-C motif chemokine 9	-0.078	0.044
CASP-8	Q14790	Caspase-8	-0.143	0.044
IL-22 RA1	Q8N6P7	Interleukin-22 receptor subunit alpha-1	-0.417	0.047
FGF-21	Q9NSA1	Fibroblast growth factor 21	-0.217	0.002
CXCL1	P09341	Growth-regulated alpha protein	0.057	0.024
OSM	P13725	Oncostatin M	0.091	0.040
CXCL5	P42830	C-X-C motif chemokine 5	0.055	0.037

Log_2_ fold-change (FC) between the Dys-NC and NG-NC groups, calculated based on arithmetic mean values. P values were determined using unpaired Student’s t-test. Proteins with statistically significant differences are presented (p < 0.05).

**Table 4:** ROC curve analysis and cutoff NPX between paired groups.

Variables	Area	P value	95% CI		Cutoff NPX
			Lower limit	Upper limit	
NG-NC VS. Dys-NC					
CCL3	0.338	0.045	0.185	0.491	4.659
CX3CL1	0.182	0.000	0.072	0.292	1.741
FGF-21	0.216	0.001	0.074	0.358	1.648
CXCL1	0.673	0.040	0.529	0.817	10.477
CCL3+CX3CL1	0.863	0.000	0.761	0.964	NA
CCL3+FGF-21	0.793	0.000	0.663	0.924	NA
CCL3+CXCL1	0.733	0.006	0.600	0.866	NA
CX3CL1+FGF-21	0.835	0.000	0.726	0.944	NA
CX3CL1+CXCL1	0.863	0.000	0.767	0.958	NA
FGF-21+CXCL1	0.836	0.000	0.730	0.943	NA
CCL3+CX3CL1+FGF-21	0.864	0.000	0.766	0.963	NA
CCL3+CX3CL1+CXCL1	0.892	0.000	0.806	0.977	NA
CCL3+FGF-21+CXCL1	0.867	0.000	0.775	0.960	NA
CX3CL1+FGF-21+CXCL1	0.880	0.000	0.791	0.969	NA
*CCL3+CX3CL1+FGF-21+CXCL1*	*0.903*	*0.000*	*0.826*	*0.980*	*NA*
Dys-NC VS. Dys-VCI					
TNFB	0.653	0.021	0.531	0.776	5.067
IL10	0.774	0.000	0.664	0.883	4.371
IL-12B	0.647	0.028	0.521	0.772	6.444
LIF-R	0.642	0.034	0.517	0.766	3.189
CX3CL1	0.676	0.008	0.555	0.798	3.424
TNFB+IL10	0.783	0.000	0.677	0.890	NA
TNFB+IL-12B	0.669	0.011	0.549	0.790	NA
TNFB+LIF-R	0.668	0.012	0.547	0.789	NA
TNFB+CX3CL1	0.695	0.003	0.578	0.812	NA
IL10+IL-12B	0.776	0.000	0.667	0.884	NA
IL10+LIF-R	0.778	0.000	0.669	0.887	NA
IL10+CX3CL1	0.786	0.000	0.680	0.892	NA
IL-12B+LIF-R	0.688	0.005	0.567	0.809	NA
IL-12B+CX3CL1	0.672	0.010	0.549	0.794	NA
LIF-R+CX3CL1	0.695	0.003	0.573	0.817	NA
TNFB+IL10+IL-12B	0.782	0.000	0.676	0.888	NA
TNFB+IL10+LIF-R	0.787	0.000	0.681	0.892	NA
TNFB+IL10+CX3CL1	0.797	0.000	0.693	0.900	NA
TNFB+IL-12B+LIF-R	0.693	0.004	0.575	0.811	NA
TNFB+IL-12B+CX3CL1	0.701	0.003	0.585	0.818	NA
TNFB+LIF-R+CX3CL1	0.705	0.002	0.588	0.822	NA
IL10+IL-12B+LIF-R	0.776	0.000	0.668	0.885	NA
IL10+IL-12B+CX3CL1	0.785	0.000	0.678	0.891	NA
IL10+LIF-R+CX3CL1	0.783	0.000	0.676	0.890	NA
IL-12B+LIF-R+CX3CL1	0.714	0.001	0.596	0.831	NA
TNFB+IL10+IL-12B+LIF-R	0.787	0.000	0.682	0.892	NA
*TNFB+IL10+IL-12B+CX3CL1*	*0.799*	*0.000*	*0.697*	*0.902*	*NA*
TNFB+IL10+LIF-R+CX3CL1	0.795	0.000	0.692	0.898	NA
TNFB+IL-12B+LIF-R+CX3CL1	0.718	0.001	0.604	0.832	NA
IL10+IL-12B+LIF-R+CX3CL1	0.783	0.000	0.676	0.890	NA
TNFB+IL10+IL-12B+LIF-R+CX3CL1	0.797	0.000	0.694	0.901	NA
NG-NC VS. Dys-VCI					
STAMBP	0.276	0.007	0.121	0.432	3.261
LIF-R	0.668	0.042	0.527	0.809	3.313
CASP-8	0.300	0.016	0.149	0.451	2.156
SIRT-2	0.300	0.016	0.139	0.461	1.065
STAMBP+LIF-R	0.793	0.000	0.668	0.918	NA
STAMBP+CASP-8	0.729	0.006	0.576	0.882	NA
STAMBP+SIRT-2	0.742	0.003	0.588	0.895	NA
LIF-R+CASP-8	0.761	0.002	0.630	0.892	NA
LIF-R+SIRT-2	0.781	0.001	0.650	0.911	NA
CASP-8+SIRT-2	0.697	0.017	0.539	0.856	NA
STAMBP+LIF-R+CASP-8	0.794	0.000	0.669	0.920	NA
STAMBP+LIF-R+SIRT-2	0.806	0.000	0.689	0.922	NA
STAMBP+CASP-8+SIRT-2	0.740	0.004	0.586	0.895	NA
LIF-R+CASP-8+SIRT-2	0.783	0.001	0.655	0.911	NA
*STAMBP+LIF-R+CASP-8+SIRT-2*	*0.808*	*0.000*	*0.694*	*0.923*	*NA*

NG-NC, normal glucose with normal cognitive function group; Dys-NC, dysglycemia with normal cognitive function group; Dys-VCI, dysglycemia with vascular cognitive impairment group.

**Figure 3:**
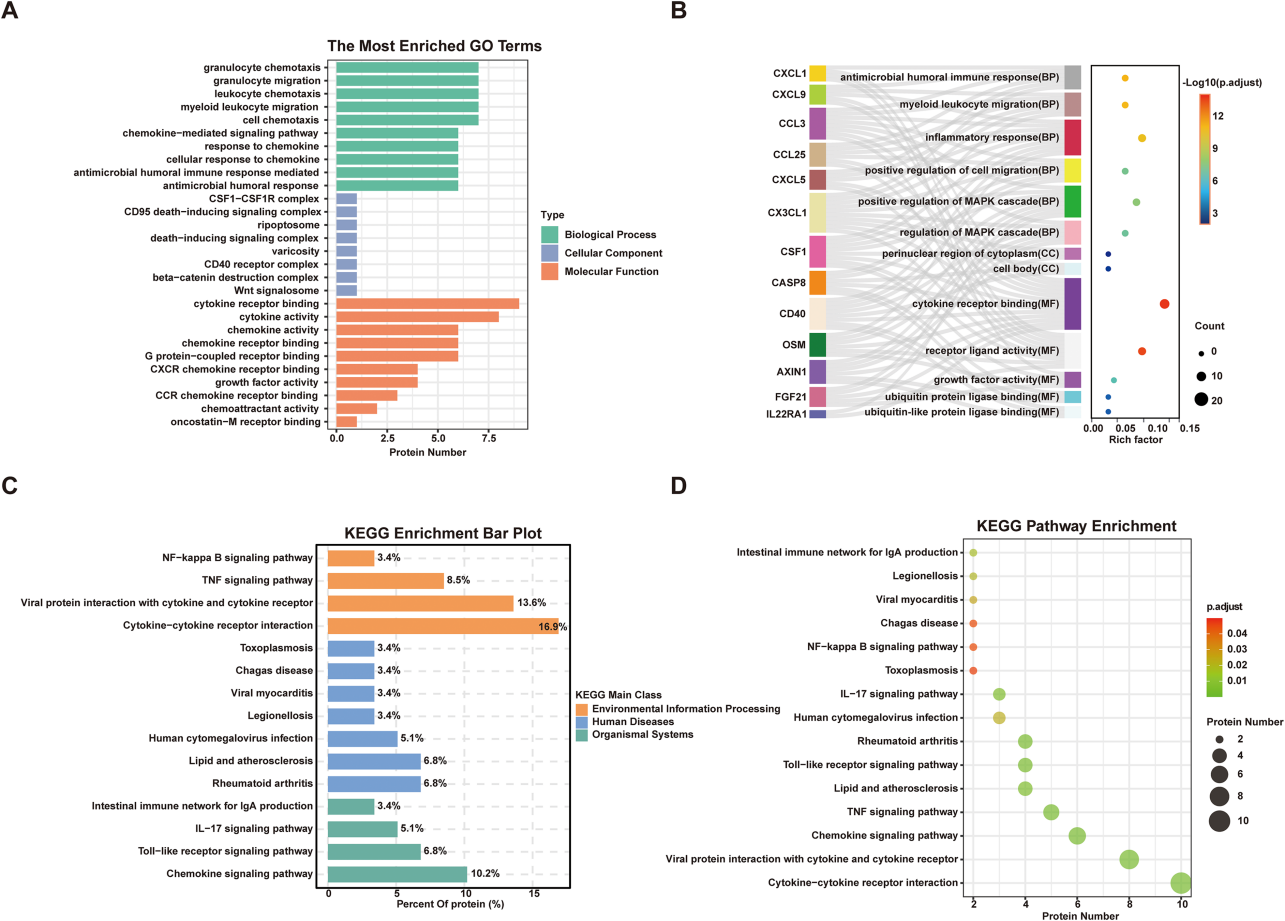
Gene ontology (GO) terms and Kyoto encyclopedia of genes and genomes (KEGG) pathway analysis of significantly dysregulated inflammatory proteins between NG-NC and Dys-NC. **(A, B)** GO term enrichment analysis of differentially expressed inflammatory proteins between NG-NC and Dys-NC. **(C, D)** KEGG pathway enrichment analysis of significantly altered proteins, highlighting associated signaling pathways between NG-NC and Dys-NC. (P-values were adjusted for multiple testing using the Benjamini-Hochberg procedure (FDR); only terms and pathways with adjusted P < 0.05 are shown. NG-NC, normal glucose with normal cognitive function group; Dys-NC, dysglycemia with normal cognitive function group.).

We conducted GO and KEGG enrichment analyses to explore the potential roles of DEPs. GO analysis revealed significant enrichment in biological processes related to immune response and cell migration, including granulocyte and leukocyte chemotaxis, chemokine-mediated signaling, and cellular response to chemokines (**Figure 3A**). In terms of molecular function, the DEPs were mainly associated with cytokine receptor binding and receptor ligand activity (**Figure 3B**). KEGG analysis identified core inflammatory regulatory pathways dominated by cytokine-cytokine receptor interaction, viral protein interaction with cytokine and cytokine receptor, TNF signaling, and chemokine signaling pathway (**Figure 3C**). Moreover, pathways linked to infectious diseases (toxoplasmosis and viral myocarditis) and autoimmune conditions (rheumatoid arthritis), as well as the intestinal immune network for IgA production, were significantly enriched (**Figure 3D**). These findings indicate that DEPs mediate inflammation and immune regulation in dysglycemia via these key pathways.

### Comparison between the Dys-NC and Dys-VCI groups

**Figure 4:**
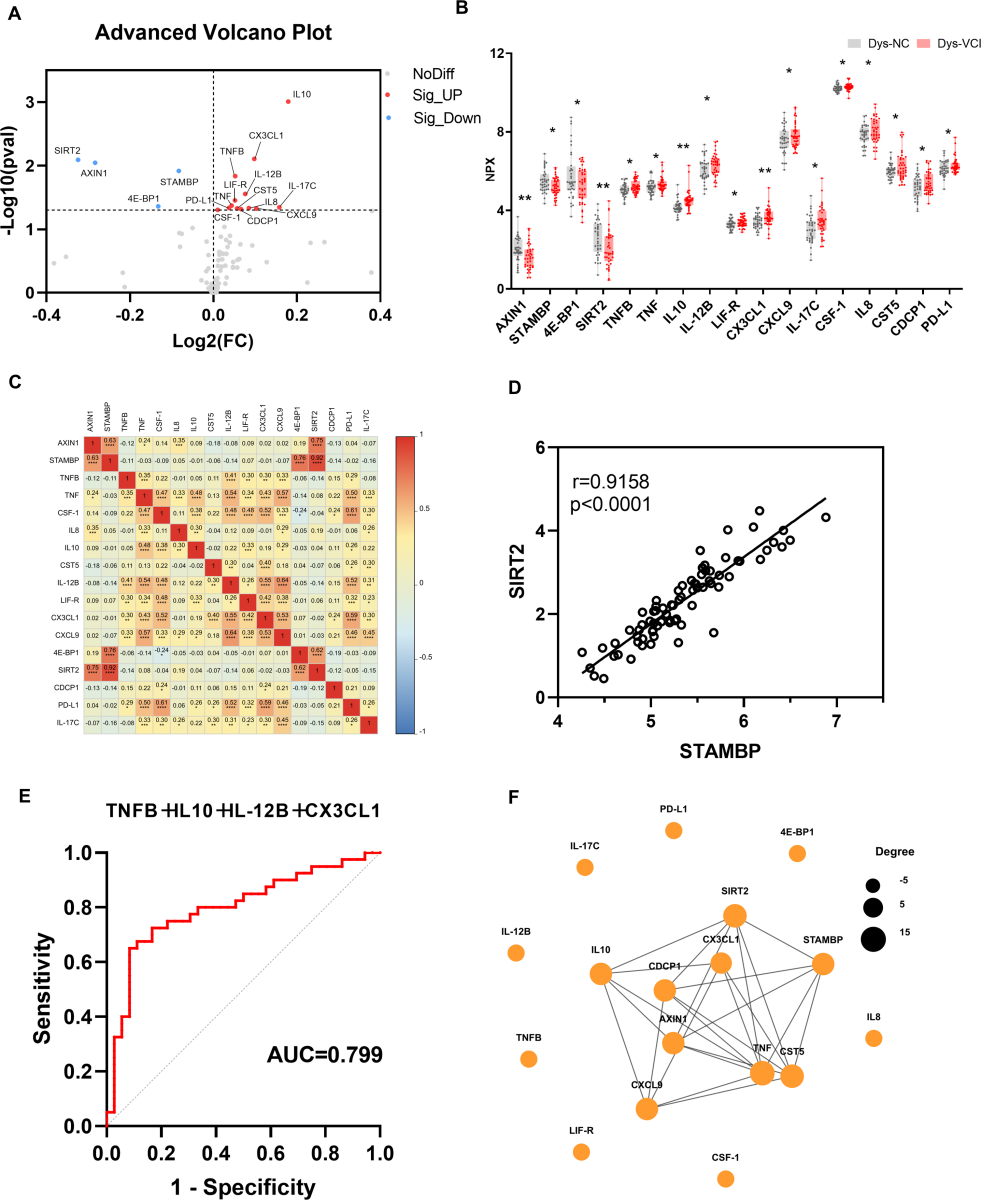
Comparison of the inflammation-related proteins between Dys-NC and Dys-VCI groups. **(A)** Volcano plot illustrating the differential expression of cytokines between the Dys-NC and Dys-VCI groups analyzed by unpaired Student’s t-test. Proteins significantly upregulated in the Dys-VCI group relative to Dys-NC are highlighted in red, whereas those downregulated are shown in blue. **(B)** Box plot representing differentially expressed proteins in the Dys-VCI group compared to the Dys-NC group with data presented as mean ± standard deviation (SD) and statistical comparisons performed using unpaired Student’s t-test. **(C)** Heatmap visualizing correlation analysis among DEPs calculated using Pearson correlation coefficients. The color scale indicates the strength and direction of correlations, with red indicating positive correlations and blue indicating negative correlations. **(D)** Scatter plot depicting the correlation between SIRT2 and STAMBP expression levels in the study population with Pearson correlation coefficient r and P value reported. **(E)** ROC curve showing the diagnostic performance of the four-protein panel (TNFB, IL10, IL-12B, and CX3CL1) in distinguishing Dys-VCI from Dys-NC; **(F)** The PPI network between all the DEPs was established by using the STRING database. (Dys-NC, dysglycemia with normal cognitive function group; Dys-VCI, dysglycemia with vascular cognitive impairment group. * <0.05; **<0.01; ***<0.001).

Then, we conducted a subgroup analysis and assessed the difference in expression levels of inflammation-related proteins between Dys-NC and Dys-VCI groups by Olink analysis, as presented in **Table 2**. As shown in **Table 5**, we found 17 differentially expressed inflammation related proteins in Dys-VCI group compared with Dys-NC group, including 13 up-regulated DEPs (TNFB, TNF, IL10, IL-12B, LIF-R, CX3CL1, CXCL9, IL-17C, CSF-1, IL8, CST5, CDCP1, PD-L1) and 4 down-regulated DEPs (AXIN1, STAMBP, 4E-BP1, SIRT2). Volcano plot and box plot showed DEPs between Dys-NC and Dys-VCI groups in **Figures 4A, B**. We conducted correlation analyses among these DEPs and illustrated the findings via a heatmap (**Figure 4C**). The most significant correlation was found between SIRT2 and STAMBP, with an r value of 0.92 and a p-value less than 0.0001 (**Figure 4D**). Through binary logistic regression, we pinpointed five DEPs that might serve as diagnostic markers: TNFB, IL10, IL-12B, LIF-R, and CX3CL1. Their diagnostic performance was evaluated using ROC curve analysis, with AUC values, 95% confidence intervals, and p-values summarized in **Table 4**. Based on the Youden index, the optimal cutoff NPX values were determined to be 5.067 for TNFB, 4.371 for IL10, 6.444 for IL-12B, 3.189 for LIF-R, and 3.424 for CX3CL1 (**Table 4**). Particularly, the combination of four DEPs (TNFB, IL10, IL-12B, and CX3CL1) achieved the highest diagnostic accuracy, yielding an AUC of 0.799 (95% CI, 0.697-0.902; p < 0.001) (**Table 4**, **Figure 4E**). Additionally, to explore potential interactions among the DEPs, the PPI network was constructed, revealing AXIN1, SIRT2 and TNF as core hub proteins with high degrees of connectivity, indicating their key regulatory roles in the network (**Figure 4F**).

**Table 5:** Significantly changed serum inflammatory proteins between the Dys-NC group and the Dys-VCI group.

Protein symbol	Uniprot ID	Name	Log_2_ FC	P value
AXIN1	O15169	Axin-1	-0.283	0.009
STAMBP	O95630	STAM-binding protein	-0.083	0.012
4E-BP1	Q13541	Eukaryotic translation initiation factor 4E-binding protein 1	-0.132	0.044
SIRT2	Q8IXJ6	NAD-dependent protein deacetylase sirtuin-2	-0.324	0.008
TNFB	P01374	Lymphotoxin-alpha	0.052	0.015
TNF	P01375	Tumor necrosis factor	0.044	0.042
IL10	P22301	Interleukin-10	0.180	0.000
IL-12B	P29460	Interleukin-12 subunit beta	0.076	0.028
LIF-R	P42702	Leukemia inhibitory factor receptor	0.052	0.035
CX3CL1	P78423	Fractalkine	0.098	0.008
CXCL9	Q07325	C-X-C motif chemokine 9	0.063	0.047
IL-17C	Q9P0M4	Interleukin-17C	0.158	0.045
CSF-1	P09603	Macrophage colony-stimulating factor 1	0.010	0.049
IL8	P10145	Interleukin-8	0.055	0.046
CST5	P28325	Cystatin-D	0.056	0.047
CDCP1	Q9H5V8	CUB domain-containing protein 1	0.066	0.048
PD-L1	Q9NZQ7	Programmed cell death 1 ligand 1	0.038	0.046

Log_2_ fold-change (FC) between the Dys-VCI and Dys-NC groups, calculated based on arithmetic mean values. P values were determined using unpaired Student’s t-test. Proteins with statistically significant differences are presented (p < 0.05).

**Figure 5:**
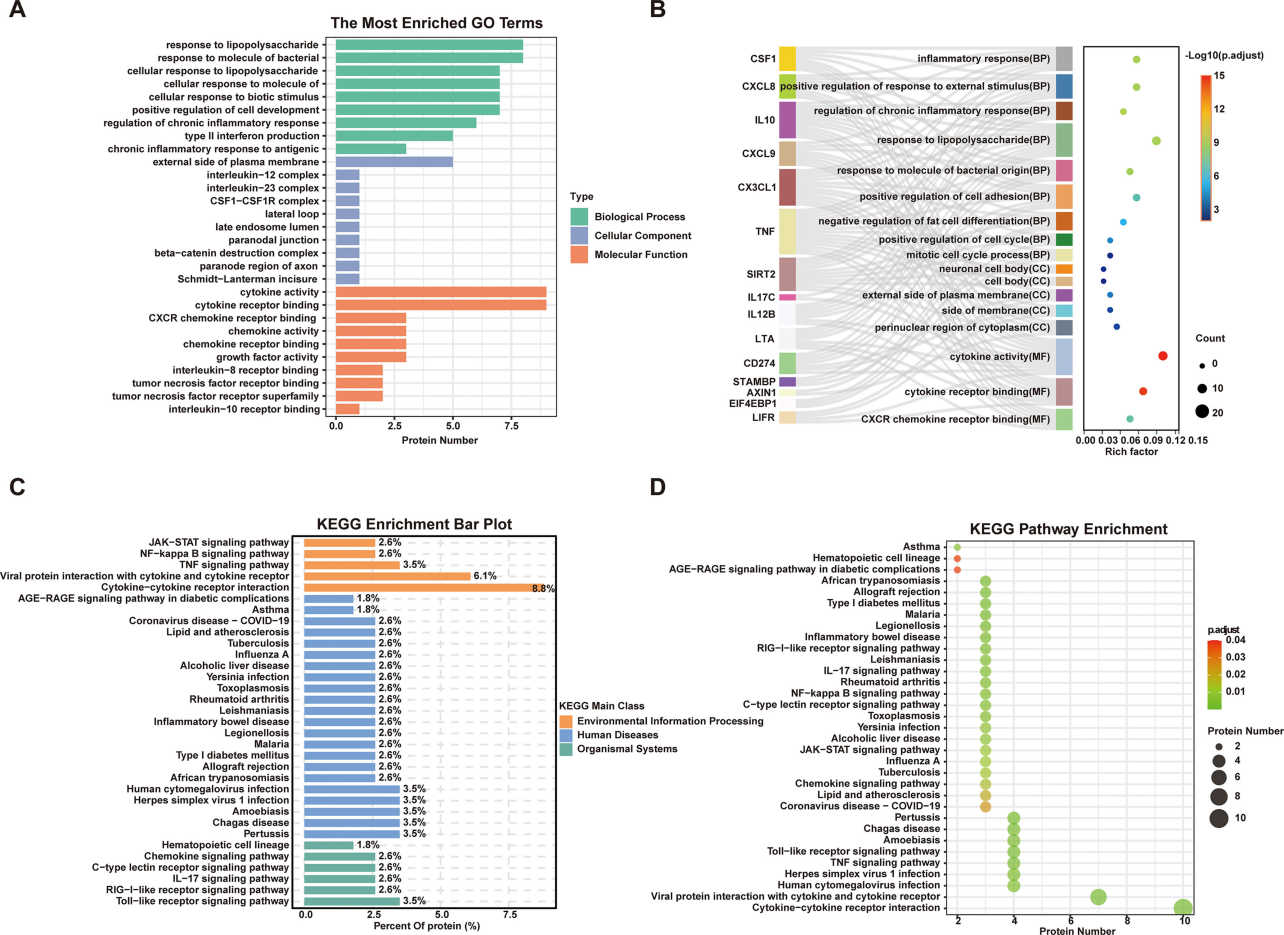
Gene ontology (GO) terms and Kyoto encyclopedia of genes and genomes (KEGG) pathway analysis of significantly dysregulated inflammatory proteins between Dys-NC and Dys-VCI. **(A, B)** GO term enrichment analysis of differentially expressed inflammatory proteins between Dys-NC and Dys-VCI. **(C, D)** KEGG pathway enrichment analysis of significantly altered proteins, highlighting associated signaling pathways between Dys-NC and Dys-VCI. (P-values were adjusted for multiple testing using the Benjamini-Hochberg procedure (FDR); only terms and pathways with adjusted P < 0.05 are shown. Dys-NC, dysglycemia with normal cognitive function group; Dys-VCI, dysglycemia with vascular cognitive impairment group.).

GO and KEGG enrichment analyses were carried out to elucidate the potential biological roles of DEPs. Functional annotation revealed that differentially expressed proteins in the Dys-VCI group were predominantly enriched in inflammatory response, leukocyte activation, and immune response pathways in the biological process category (**Figures 5A, B**). Consistent with these findings, KEGG pathway analysis further highlighted the significant enrichment of DEPs in cytokine-cytokine receptor interaction, viral protein interaction with cytokine and cytokine receptor, TNF signaling pathway, and NF-κB signaling pathway; additionally, pathways linked to diabetic complications (AGE-RAGE signaling), infectious diseases and autoimmune conditions (rheumatoid arthritis) were also enriched (**Figures 5C, D**). These data suggest that in the context of dysglycemia, vascular cognitive impairment is accompanied by a marked activation of systemic inflammatory responses.

### Comparison between the NG-NC and Dys-VCI groups

**Figure 6:**
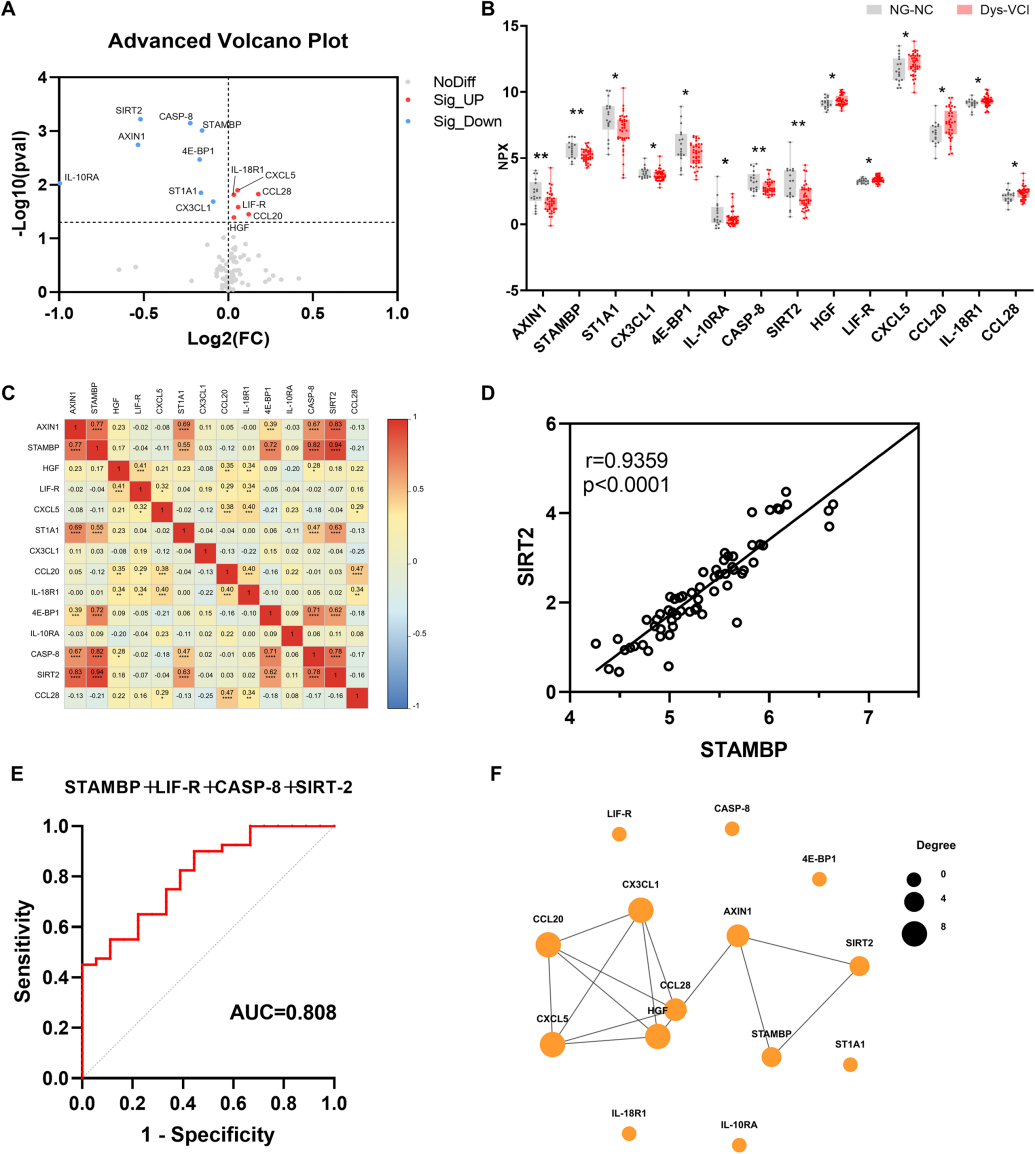
Comparison of the inflammation-related proteins between NG-NC and Dys-VCI groups. **(A)** Volcano plot illustrating the differential expression of cytokines between the Dys-NC and NG-NC groups analyzed by unpaired Student’s t-test. Proteins significantly upregulated in the Dys-VCI group relative to NG-NC are highlighted in red, whereas those downregulated are shown in blue. **(B)** Box plot representing differentially expressed proteins in the Dys-VCI group compared to the NG-NC group with data presented as mean ± standard deviation (SD) and statistical comparisons performed using unpaired Student’s t-test. **(C)** Heatmap visualizing correlation analysis among DEPs calculated using Pearson correlation coefficients. The color scale indicates the strength and direction of correlations, with red indicating positive correlations and blue indicating negative correlations. **(D)** Scatter plot depicting the correlation between SIRT2 and STAMBP expression levels in the study population with Pearson correlation coefficient r and P value reported. **(E)** ROC curve showing the diagnostic performance of the four-protein panel (STAMBP, LIF-R, CASP-8, and SIRT-2) in distinguishing Dys-VCI from NG-NC. **(F)** The PPI network between all the DEPs was established by using the STRING database. (NG-NC, normal glucose with normal cognitive function group; Dys-VCI, dysglycemia with vascular cognitive impairment group. * <0.05; **<0.01).

We subsequently performed a subgroup analysis to evaluate the differences in expression levels of inflammation-related proteins between the NG-NC and Dys-VCI groups, as detailed in **Table 2**. As presented in **Table 6**, total of 14 inflammation-associated proteins were identified as differentially expressed in the Dys-VCI group compared with NG-NC group. Among these, 6 were upregulated (HGF, LIF-R, CXCL5, CCL20, IL-18R1, CCL28), while 8 were downregulated (AXIN1, STAMBP, ST1A1, CX3CL1, 4E-BP1, IL-10RA, CASP-8, SIRT2). The differential expression of these proteins is visually represented through a volcano plot and box plot in **Figures 6A, B**, respectively. To further explore relationships among the DEPs, correlation analyses were conducted and visualized using a heatmap (**Figure 6C**). The strongest positive correlation was observed between SIRT2 and STAMBP, with a correlation coefficient of 0.94 and a p-value < 0.0001 (**Figure 6D**). Using binary logistic regression analysis, four DEPs, STAMBP, LIF-R, CASP-8, and SIRT-2, were identified as potential diagnostic biomarkers. Their diagnostic performance was assessed by ROC curve analysis, and the AUC values, 95% confidence intervals, and corresponding p-values are summarized in **Table 4**. Based on the Youden index, the optimal cutoff NPX values were determined as follows: 0.276 for STAMBP, 0.668 for LIF-R, 0.300 for CASP-8, and 0.300 for SIRT-2. Notably, the combination of these four DEPs demonstrated the highest diagnostic accuracy, achieving an AUC of 0.808 (95% CI: 0.694-0.923; p < 0.001), as shown in **Table 4** and **Figure 6E**. Furthermore, to investigate potential molecular interactions among the DEPs, a PPI network was constructed, identifying CX3CL1, CCL20, CXCL5, and HGF as forming a tightly interconnected core hub cluster that serves as key interaction nodes in the network (**Figure 6F**). These findings suggest their critical regulatory roles in the inflammatory processes associated with Dys-VCI.

**Table 6:** Significantly changed serum inflammatory proteins between the NG-NC group and the Dys-VCI group.

Protein symbol	Uniprot ID	Name	Log_2_ FC	P value
AXIN1	O15169	Axin-1	-0.534	0.004
STAMBP	O95630	STAM-binding protein	-0.156	0.001
ST1A1	P50225	Sulfotransferase 1A1	-0.162	0.044
CX3CL1	P78423	Fractalkine	-0.090	0.040
4E-BP1	Q13541	Eukaryotic translation initiation factor 4E-binding protein 1	-0.169	0.034
IL-10RA	Q13651	Interleukin-10 receptor subunit alpha	-1.096	0.034
CASP-8	Q14790	Caspase-8	-0.225	0.007
SIRT2	Q8IXJ6	NAD-dependent protein deacetylase sirtuin-2	-0.520	0.006
HGF	P14210	Hepatocyte growth factor	0.033	0.041
LIF-R	P42702	Leukemia inhibitory factor receptor	0.059	0.036
CXCL5	P42830	C-X-C motif chemokine 5	0.055	0.046
CCL20	P78556	C-C motif chemokine 20	0.121	0.045
IL-18R1	O13478	Interleukin-18 receptor 1	0.033	0.035
CCL28	Q9NRJ3	C-C motif chemokine 28	0.177	0.045

Log_2_ fold-change (FC) between the Dys-VCI and NG-NC groups, calculated based on arithmetic mean values. P values were determined using unpaired Student’s t-test. Proteins with statistically significant differences are presented (p < 0.05).

**Figure 7:**
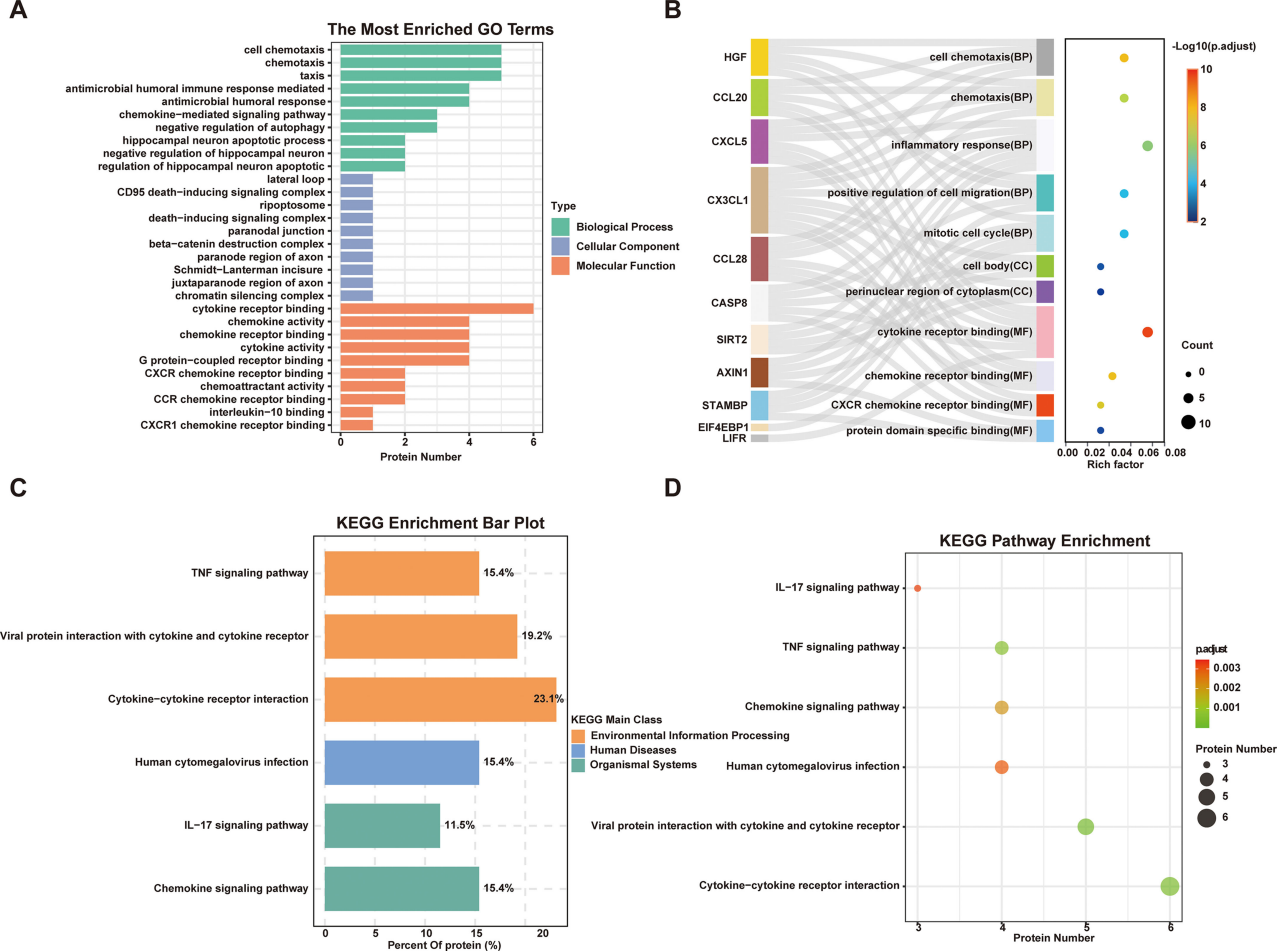
Gene ontology (GO) terms and Kyoto encyclopedia of genes and genomes (KEGG) pathway analysis of significantly dysregulated inflammatory proteins between NG-NC and Dys-VCI. **(A, B)** GO term enrichment analysis of differentially expressed inflammatory proteins between NG-NC and Dys-VCI. **(C, D)** KEGG pathway enrichment analysis of significantly altered proteins, highlighting associated signaling pathways between NG-NC and Dys-VCI. (P-values were adjusted for multiple testing using the Benjamini-Hochberg procedure (FDR); only terms and pathways with adjusted P < 0.05 are shown. NG-NC, normal glucose with normal cognitive function group; Dys-VCI, dysglycemia with vascular cognitive impairment group.).

To uncover the functional implications of DEPs, we conducted GO and KEGG pathway enrichment analyses. **Figures 7A, B** present the GO enrichment results, showing that the differentially expressed proteins are predominantly involved in biological processes such as cell chemotaxis, chemotaxis, and taxis. In terms of molecular functions, these DEPs were significantly enriched in cytokine receptor binding and chemokine activity, indicating their involvement in immune and inflammatory signaling. Key proteins including CX3CL1, CXCL5 and CCL28 were found to be strongly associated with multiple enriched pathways, suggesting their central roles in disease-related mechanisms. **Figures 7C, D** show the KEGG pathway analysis, which revealed significant enrichment in cytokine-cytokine receptor interaction, viral protein interaction with cytokine and cytokine receptor, TNF signaling, chemokine signaling, IL-17 signaling pathways, as well as human cytomegalovirus infection-related pathways. These findings collectively highlight the critical involvement of inflammation-related pathways and immune dysregulation in the pathophysiology of Dys-VCI.

## Discussion

VCI is defined as cognitive dysfunction primarily caused by cerebrovascular disease and its risk factors (6). Currently, VCI is estimated to affect approximately 20 million individuals worldwide, with an increasing trend in the coming decades, particularly in low-to-middle-income countries (1). DM is the most prevalent metabolic disorder and a major risk factor for VCI, and its prevalence rises with advancing age (28). Diabetes leads to cerebral atherosclerosis, causing significant structural and functional alterations in cerebral vasculature and reduced cerebral blood flow (29–31). Furthermore, hyperglycemia accelerates the formation of advanced glycation end products (AGEs), which are closely linked to amyloid deposition, tau pathology, and increased oxidative stress, which are key mechanisms implicated in the pathogenesis of VCI (32, 33). Emerging evidence highlights a critical role of systemic inflammation in the pathogenesis of both DM and VCI (34–36). Chronic low-grade inflammation is increasingly recognized as critical mechanism linking metabolic dysregulation to neurodegenerative processes (37, 38). However, comprehensive profiling of inflammatory mediators across different metabolic and cognitive stages remains limited. Serum is noted as a cost-effective and accessible source for biomarker discovery (39). Serum protein content reflects systemic inflammation and potential interactions between peripheral immunity and CNS pathology, making it valuable for identifying early disease markers and therapeutic responses (40, 41). In this study, we utilized the Olink proteomics platform, a high-throughput, multiplex immunoassay technology with exceptional sensitivity and specificity, to characterize serum inflammatory protein profiles in three well-defined clinical groups: NG-NC (normal glucose with normal cognition), Dys-NC (dysglycemia with normal cognition), and Dys-VCI (dysglycemia with vascular cognitive impairment). This targeted proteomic approach aimed to identify stage-specific inflammatory patterns and key proteins that may serve as biomarkers or therapeutic links between dysglycemia, neuroinflammation, and cognitive decline.

In this study, comparative analysis among these groups uncovered distinct patterns of protein expression, reflecting progressive immune activation during the transition from normoglycemia to cognitive impairment. In the early metabolic stage (NG-NC vs Dys-NC), 13 DEPs were identified, with 3 upregulated and 10 downregulated. Notably, upregulated proteins such as CXCL1, OSM, and CXCL5 are involved in pro-inflammatory signaling, leukocyte recruitment, and epithelial-mesenchymal transition (42–44). In addition, downregulated proteins like AXIN1, FGF-21, and CX3CL1 are linked to anti-inflammatory pathways, neuronal protection, and immune homeostasis. These findings suggest that systemic inflammation is already activated at the prediabetic stage, even before cognitive symptoms begin. When comparing Dys-NC with Dys-VCI, we observed a further amplification of inflammatory responses. A shift toward a pro-inflammatory state was evident in the Dys-VCI group, with significant upregulation of cytokines such as TNFB, TNF, IL-12B, CXCL9, IL-17C, and CSF-1 (45, 46). In contrast, endogenous anti-inflammatory mediators, including AXIN1 and SIRT2, were downregulated, suggesting an imbalance in immune regulation associated with cognitive decline in the context of dysglycemia. Interestingly, several proteins showed consistent changes across disease stages. For instance, AXIN1 was consistently downregulated across all three comparisons, suggesting its involvement in early metabolic-neuroimmune dysregulation and sustained CNS imbalance. As a key scaffold protein in the β-catenin destruction complex, AXIN1 reduction may lead to aberrant Wnt/β-catenin activation and contribute to neuroinflammatory processes (47, 48). AXIN1 also suppresses NF-κB signaling to maintain immune homeostasis, its progressive loss likely exacerbates chronic inflammation and neuronal damage (49, 50). Similarly, CX3CL1 was downregulated in both the Dys-NC and Dys-VCI groups compared with NG-NC, suggesting its potential role as an early indicator of neuroinflammation and immune dysregulation. Notably, when comparing Dys-VCI with Dys-NC, CX3CL1 was upregulated, indicating a stage-specific modulation. As a key mediator of neuron-microglia communication, CX3CL1 has been implicated in both maintaining CNS homeostasis and contributing to inflammatory responses under pathological conditions, highlighting its dual functional role in metabolic-cognitive interactions (51, 52). STAMBP, a deubiquitinating enzyme of the Jab1/MPN family, specifically removes K63-linked ubiquitin chains and facilitates substrate degradation via the endosome-lysosome pathway (53). Its downregulation may impair proteostasis and contribute to neurodegeneration (54). Notably, STAMBP knockout mice exhibit hippocampal neuronal loss, postnatal growth retardation, and early lethality (55), highlighting its essential role in neural survival. SIRT2, a cytoplasmic sirtuin family member, plays a key role in regulating inflammation and oxidative stress, its downregulation may promote neuroinflammation and neuronal damage (56, 57). Accumulating evidence links SIRT2 dysregulation to neurodegenerative diseases such as AD, depression, and stroke (58). The progressive reduction of SIRT2 in Dys-VCI suggests that SIRT2 may represent a molecular link between metabolic dysfunction and cognitive decline.

Functional enrichment analyses revealed a progressive involvement of inflammatory and immune-related pathways across the three comparison groups, suggesting a transition from early immune activation to sustained inflammation in the development of VCI. DEPs were consistently enriched in biological processes such as chemotaxis and cytokine/chemokine-related signaling, with key pathways including cytokine-cytokine receptor interaction, TNF signaling, and chemokine signaling being activated across different stages. In the metabolic impairment stage (NG-NC vs. Dys-NC), pathways related to chemokine-mediated signaling, granulocyte and leukocyte chemotaxis, and early inflammatory responses were prominent, reflecting an initial activation of systemic immune surveillance (59). As the condition progressed to cognitive impairment (Dys-NC vs. Dys-VCI), pro-inflammatory pathways became further amplified, with TNF and NF-κB signaling pathways being significantly enriched, indicating a shift toward sustained immune activation. Molecular function analysis highlighted the persistent importance of cytokine receptor binding and receptor ligand activity throughout disease progression, emphasizing the central role of cytokine-chemokine interactions in the transition from metabolic dysfunction to cognitive decline (60). Notably, several DEPs such as TNF, SIRT2, and CX3CL1 were involved in multiple enriched pathways, indicating their potential roles in linking metabolic stress with immune dysregulation and cognitive decline (61). PPI network analysis highlighted CX3CL1and CXCL5 as hub nodes, underscoring their regulatory roles in immune homeostasis and neuroprotection (51, 58). These proteins warrant further mechanistic investigation for their potential as therapeutic targets or early biomarkers of dysglycemia-related cognitive impairment.

To assess the translational value of our findings, we constructed logistic regression models using selected DEPs. A four-protein panel (CCL3, CX3CL1, FGF-21, and CXCL1) achieved an AUC of 0.903 for distinguishing Dys-NC from NG-NC, while another four-protein set (TNFB, IL10, IL-12B, and CX3CL1) demonstrated an AUC of 0.799 for identifying Dys-VCI among dysglycemic individuals. These results indicate that combinations of inflammatory proteins may serve as promising non-invasive biomarkers for early detection and risk stratification in at-risk populations (62). Our findings were derived from a clinically well-characterized cohort using a targeted proteomic approach, allowing us to capture distinct inflammatory patterns across different metabolic and cognitive stages (63). The integration of multi-omics profiling further enhances the biological relevance of the identified proteins (64). Despite these advantages, the current study has several limitations. First, our findings regarding stage-specific inflammatory patterns should be interpreted as exploratory. The absence of multivariable adjustment for medications and other vascular risk factors, combined with the cross-sectional design and limited sample size, limits the robustness of observed associations. Although our sample size is comparable to similar Olink studies, it may have limited statistical power and increased Type II error risk. Therefore, our nominally significant results (p < 0.05) require replication in larger, independent, and preferably longitudinal cohorts. Although the three groups were well-matched in blood pressure and lipid profiles, suggesting comparable medication exposure, we lack detailed information on specific drug regimens beyond our exclusion criteria. Future prospective studies with comprehensive medication records are needed to fully elucidate potential drug-related confounding effects. The relatively small sample size precluded conventional modeling-validation splitting for ROC analysis; future larger-scale studies will validate these findings using independent modeling and validation cohorts. Additionally, while differentially expressed proteins were identified across the three groups, our statistical analyses did not precisely define specific biomarkers or combined panels for VCI diagnosis. The lack of functional validation and imaging integration limits clinical utility and mechanistic insights. In our future work, we plan to expand the sample size and integrate neuroimaging data to validate the identified protein signatures, aiming to refine biomarker screening and develop more robust diagnostic panels for VCI. While our models focused on pairwise comparisons, advanced multi-class bioinformatics approaches could better capture changes across all three groups. Future studies with larger sample sizes should implement machine learning algorithms to develop integrated classification models that can simultaneously distinguish NG-NC, Dys-NC, and Dys-VCI individuals.

## Conclusions

In conclusion, we identified a panel of inflammatory proteins dynamically altered across the metabolic-cognitive continuum. A four-protein signature consisting of CCL3, CX3CL1, FGF-21, and CXCL1 showed strong discriminatory power for detecting early dysglycemia-related immune dysregulation. Another set including TNFB, IL10, IL-12B, and CX3CL1 demonstrated moderate predictive value for vascular cognitive impairment in individuals with dysglycemia. Functional analyses revealed that these proteins were primarily enriched in cytokine-cytokine receptor interaction, chemokine signaling, and TNF signaling. These findings suggest that systemic inflammation is progressively activated during the transition from metabolic disturbance to cognitive decline. The identified protein panels may serve as potential biomarkers for early detection or risk stratification. Targeting the associated inflammatory pathways could offer novel therapeutic strategies for preventing VCI.

## Data Availability

The datasets presented in this study can be found in online repositories. The names of the repository/repositories and accession number(s) can be found in the article/supplementary material.

## References

[1] Mok VCT Cai Y Markus HS . Vascular cognitive impairment and dementia: mechanisms, treatment, and future directions. Int J Stroke. (2024) 19:838–856. doi: 10.1177/17474930241279888. PMID: 39283037 PMC11490097

[2] Hosoki S Hansra GK Jayasena T Poljak A Mather KA Catts VS . Molecular biomarkers for vascular cognitive impairment and dementia. Nat Rev Neurol. (2023) 19(12):737–753. doi: 10.1038/s41582-023-00884-1. PMID: 37957261

[3] Chang Wong E Chang Chui H . Vascular cognitive impairment and dementia. Continuum (Minneap Minn). (2022) 28:750–780. doi: 10.1212/con.0000000000001124. PMID: 35678401 PMC9833847

[4] Pantoni L Fierini F Poggesi A . Impact of cerebral white matter changes on functionality in older adults: an overview of the LADIS study results and future directions. Geriatr Gerontol Int. (2015) 15:10–16. doi: 10.1111/ggi.12665. PMID: 26671152

[5] Benisty S Gouw AA Porcher R Madureira S Hernandez K Poggesi A . Location of lacunar infarcts correlates with cognition in a sample of non-disabled subjects with age-related white-matter changes: the LADIS study. J Neurol Neurosurg Psychiatry. (2009) 80(5):478–483. doi: 10.1136/jnnp.2008.160440. PMID: 19211595

[6] Rundek T Tolea M Ariko T Fagerli EA Camargo CJ . Vascular cognitive impairment (VCI). Neurotherapeutics. (2022) 19(1):68–88. doi: 10.1007/s13311-021-01170-y. PMID: 34939171 PMC9130444

[7] Sachdev PS Bentvelzen AC Gustafson D Hansra GK Hosoki S Jiang J . Vascular cognitive impairment and dementia: clinical features, neuropathology, and biomarkers. J Am Coll Cardiol. (2026) 87(1):52–76. doi: 10.1016/j.jacc.2025.11.008 PMC13553213 41498479

[8] Rajeev V Chai YL Poh L Selvaraji S Fann DY Jo DG . Chronic cerebral hypoperfusion: a critical feature in unravelling the etiology of vascular cognitive impairment. Acta Neuropathol Commun. (2023) 11(1):93. doi: 10.1186/s40478-023-01590-1. PMID: 37309012 PMC10259064

[9] Wu LY Chai YL Cheah IK Chia RSL Hilal S Arumugam TV . Blood-based biomarkers of cerebral small vessel disease. Ageing Res Rev. (2024) 95:102247. doi: 10.1016/j.arr.2024.102247. PMID: 38417710

[10] Erhardt EB Adair JC Knoefel JE Caprihan A Prestopnik J Thompson J . Inflammatory biomarkers aid in diagnosis of dementia. Front Aging Neurosci. (2021) 13:717344. doi: 10.3389/fnagi.2021.717344. PMID: 34489684 PMC8416621

[11] Ma W Zhang J Xu J Feng D Wang X Zhang F . Elevated levels of serum neurofilament light chain associated with cognitive impairment in vascular dementia. Dis Markers. (2020) 2020:6612871. doi: 10.1155/2020/6612871. PMID: 33204362 PMC7652600

[12] Xu Y Lu J Li M Wang T Wang K Cao Q . Diabetes in China part 1: epidemiology and risk factors. Lancet Public Health. (2024) 9(12):e1089–e1097. doi: 10.1016/s2468-2667(24)00250-0. PMID: 39579774

[13] Ehtewish H Arredouani A El-Agnaf O . Diagnostic, prognostic, and mechanistic biomarkers of diabetes mellitus-associated cognitive decline. Int J Mol Sci. (2022) 23(11):6144. doi: 10.3390/ijms23116144. PMID: 35682821 PMC9181591

[14] American Diabetes Association Professional Practice Committee . 2. Diagnosis and classification of diabetes: standards of care in diabetes-2025. Diabetes Care. (2025) 48(1 Suppl 1):S27–S49. doi: 10.2337/dc25-S002 PMC11635041 39651986

[15] Rotonen S Auvinen J Bloigu A Härkönen P Jokelainen J Timonen M . Long-term dysglycemia as a risk factor for faster cognitive decline during aging: a 12-year follow-up study. Diabetes Res Clin Pract. (2021) 180:109045. doi: 10.1016/j.diabres.2021.109045. PMID: 34508737

[16] Cukierman-Yaffe T . Diabetes, dysglycemia and cognitive dysfunction. Diabetes Metab Res Rev. (2014) 30(5):341–345. doi: 10.1002/dmrr.2507. PMID: 24339052

[17] Biessels GJ Despa F . Cognitive decline and dementia in diabetes mellitus: mechanisms and clinical implications. Nat Rev Endocrinol. (2018) 14(10):591–604. doi: 10.1038/s41574-018-0048-7. PMID: 30022099 PMC6397437

[18] Tanne D . Impaired glucose metabolism and cerebrovascular diseases. Adv Cardiol. (2008) 45:107–113. doi: 10.1159/000115190. PMID: 18230958

[19] ElSayed NA Aleppo G Aroda VR Bannuru RR Brown FM Bruemmer D . 2. Classification and diagnosis of diabetes: standards of care in diabetes-2023. Diabetes Care. (2023) 46(Suppl 1):S19–S40. doi: 10.2337/dc23-S002. PMID: 36507649 PMC9810477

[20] Hossain MI Roulston CL Stapleton DI . Molecular basis of impaired glycogen metabolism during ischemic stroke and hypoxia. PLoS One. (2014) 9(5):e97570. doi: 10.1371/journal.pone.0097570. PMID: 24858129 PMC4032261

[21] Daulatzai MA . Cerebral hypoperfusion and glucose hypometabolism: key pathophysiological modulators promote neurodegeneration, cognitive impairment, and Alzheimer's disease. J Neurosci Res. (2017) 95(4):943–972. doi: 10.1002/jnr.23777. PMID: 27350397

[22] Lyu F Wu D Wei C Wu A . Vascular cognitive impairment and dementia in type 2 diabetes mellitus: an overview. Life Sci. (2020) 254:117771. doi: 10.1016/j.lfs.2020.117771. PMID: 32437791

[23] Tangestani Fard M Stough C . A review and hypothesized model of the mechanisms that underpin the relationship between inflammation and cognition in the elderly. Front Aging Neurosci. (2019) 11:56. doi: 10.3389/fnagi.2019.00056. PMID: 30930767 PMC6425084

[24] Sachdev PS Bentvelzen AC Kochan NA Jiang J Hosoki S et al. . Revised diagnostic criteria for vascular cognitive impairment and dementia-the VasCog-2-WSO criteria. JAMA Neurol. (2025) 82(11):1103–1112. doi: 10.1001/jamaneurol.2025.3242. PMID: 40955506 PMC12441927

[25] Chinese Stroke Association Vascular Cognitive Impairment Subcommittee . Chinese guidelines for the diagnosis and treatment of vascular cognitive impairment (2024 edition). Zhonghua Yi Xue Za Zhi. (2024) 104(31):2881–2894. doi: 10.3760/cma.j.cn112137-20240501-01024 38866700

[26] Thomann AE Berres M Goettel N Steiner LA Monsch AU . Enhanced diagnostic accuracy for neurocognitive disorders: a revised cut-off approach for the Montreal Cognitive Assessment. Alzheimers Res Ther. (2020) 12(1):39. doi: 10.1186/s13195-020-00603-8. PMID: 32264975 PMC7140337

[27] Tan JP Li N Gao J Wang LN Zhao YM Yu BC . Optimal cutoff scores for dementia and mild cognitive impairment of the Montreal Cognitive Assessment among elderly and oldest-old Chinese population. J Alzheimers Dis. (2015) 43(4):1403–1412. doi: 10.3233/jad-141278. PMID: 25147113

[28] Peila R Rodriguez BL Launer LJ . Type 2 diabetes, APOE gene, and the risk for dementia and related pathologies: the Honolulu-Asia Aging Study. Diabetes. (2002) 51(4):1256–1262. doi: 10.2337/diabetes.51.4.1256. PMID: 11916953

[29] Saczynski JS Jónsdóttir MK Garcia ME Jonsson PV Peila R Eiriksdottir G . Cognitive impairment: an increasingly important complication of type 2 diabetes: the age, gene/environment susceptibility--Reykjavik study. Am J Epidemiol. (2008) 168(10):1132–1139. doi: 10.1093/aje/kwn228. PMID: 18836152 PMC2727243

[30] Crane PK Walker R Hubbard RA Li G Nathan DM Zheng H . Glucose levels and risk of dementia. N Engl J Med. (2013) 369(6):540–548. doi: 10.1056/nejmoa1215740. PMID: 23924004 PMC3955123

[31] Tuligenga RH Dugravot A Tabák AG Elbaz A Brunner EJ Kivimäki M . Midlife type 2 diabetes and poor glycaemic control as risk factors for cognitive decline in early old age: a post-hoc analysis of the Whitehall II cohort study. Lancet Diabetes Endocrinol. (2014) 2(3):228–235. doi: 10.1016/s2213-8587(13)70192-x. PMID: 24622753 PMC4274502

[32] Badji A Youwakim J Cooper A Westman E Marseglia A . Vascular cognitive impairment - past, present, and future challenges. Ageing Res Rev. (2023) 90:102042. doi: 10.1016/j.arr.2023.102042. PMID: 37634888

[33] Roberts RO Knopman DS Cha RH Mielke MM Pankratz VS Boeve BF . Diabetes and elevated hemoglobin A1c levels are associated with brain hypometabolism but not amyloid accumulation. J Nucl Med. (2014) 55(5):759–764. doi: 10.2967/jnumed.113.132647. PMID: 24652830 PMC4011952

[34] Inoue Y Shue F Bu G Kanekiyo T . Pathophysiology and probable etiology of cerebral small vessel disease in vascular dementia and Alzheimer's disease. Mol Neurodegener. (2023) 18(1):46. doi: 10.1186/s13024-023-00640-5. PMID: 37434208 PMC10334598

[35] Jiménez-Ruiz A Aguilar-Fuentes V Becerra-Aguiar NN Roque-Sanchez I Ruiz-Sandoval JL . Vascular cognitive impairment and dementia: a narrative review. Dement Neuropsychol. (2024) 18:e20230116. doi: 10.1590/1980-5764-DN-2023-0116. PMID: 39318380 PMC11421556

[36] Rosenberg GA . Inflammation and white matter damage in vascular cognitive impairment. Stroke. (2009) 40(3 Suppl):S20–S23. doi: 10.1161/strokeaha.108.533133. PMID: 19064797 PMC2811584

[37] Zhang W Xiao D Mao Q Xia H . Role of neuroinflammation in neurodegeneration development. Signal Transduct Target Ther. (2023) 8(1):267. doi: 10.1038/s41392-023-01486-5. PMID: 37433768 PMC10336149

[38] Wilson DM 3rd Cookson MR Van Den Bosch L Zetterberg H Holtzman DM Dewachter I . Hallmarks of neurodegenerative diseases. Cell. (2023) 186(4):693–714. doi: 10.1016/j.cell.2022.12.032. PMID: 36803602

[39] Bivona G Gambino CM Lo Sasso B Scazzone C Giglio RV Agnello L . Serum vitamin D as a biomarker in autoimmune, psychiatric and neurodegenerative diseases. Diagnostics (Basel). (2022) 12(1):130. doi: 10.3390/diagnostics12010130. PMID: 35054296 PMC8774449

[40] Tao QQ Cai X Xue YY Ge W Yue L Li XY . Alzheimer's disease early diagnostic and staging biomarkers revealed by large-scale cerebrospinal fluid and serum proteomic profiling. Innovation (Camb). (2024) 5(1):100544. doi: 10.1016/j.xinn.2023.100544. PMID: 38235188 PMC10794110

[41] Gandy S Ikonomovic MD Mitsis E Elder G Ahlers ST Barth J . Chronic traumatic encephalopathy: clinical-biomarker correlations and current concepts in pathogenesis. Mol Neurodegener. (2014) 9:37. doi: 10.1186/1750-1326-9-37. PMID: 25231386 PMC4249716

[42] Korbecki J Barczak K Gutowska I Chlubek D Baranowska-Bosiacka I . CXCL1: gene, promoter, regulation of expression, mRNA stability, regulation of activity in the intercellular space. Int J Mol Sci. (2022) 23(2):792. doi: 10.3390/ijms23020792. PMID: 35054978 PMC8776070

[43] Wolf CL Pruett C Lighter D Jorcyk CL . The clinical relevance of OSM in inflammatory diseases: a comprehensive review. Front Immunol. (2023) 14:1239732. doi: 10.3389/fimmu.2023.1239732. PMID: 37841259 PMC10570509

[44] Kaltenmeier C Wang R Popp B Geller D Tohme S Yazdani HO . Role of immuno-inflammatory signals in liver ischemia-reperfusion injury. Cells. (2022) 11(14):2222. doi: 10.3390/cells11142222. PMID: 35883665 PMC9323912

[45] Chen J Doyle MF Fang Y Mez J Crane PK Scollard P . Peripheral inflammatory biomarkers are associated with cognitive function and dementia: Framingham Heart Study Offspring cohort. Aging Cell. (2023) 22(10):e13955. doi: 10.1111/acel.13955. PMID: 37584418 PMC10577533

[46] Wen B Wei S Huang D Zhang C Wang H Liu S . The connection between 91 inflammatory cytokines and frailty mediated by 1400 metabolites: an exploratory two-step Mendelian randomization analysis. Arch Gerontol Geriatr. (2025) 133:105774. doi: 10.1016/j.archger.2025.105774. PMID: 40054372

[47] Garrett S Zhang Y Xia Y Sun J . Intestinal epithelial Axin1 deficiency protects against colitis via altered gut microbiota. Engineering (Beijing). (2024) 35:241–256. doi: 10.1016/j.eng.2023.06.007. PMID: 38911180 PMC11192507

[48] Garrett S Asada MC Sun J . Axin1's mystique in manipulating microbiome amidst colitis. Gut Microbes. (2023) 15(2):2286674. doi: 10.1080/19490976.2023.2286674. PMID: 38010886 PMC10730173

[49] Bognar MK Vincendeau M Erdmann T Seeholzer T Grau M Linnemann JR . Oncogenic CARMA1 couples NF-κB and β-catenin signaling in diffuse large B-cell lymphomas. Oncogene. (2016) 35(32):4269–4281. doi: 10.1038/onc.2015.493. PMID: 26776161 PMC4981874

[50] Katoh M . Multi-layered prevention and treatment of chronic inflammation, organ fibrosis and cancer associated with canonical WNT/β-catenin signaling activation (review). Int J Mol Med. (2018) 42(2):713–725. doi: 10.3892/ijmm.2018.3689. PMID: 29786110 PMC6034925

[51] Subbarayan MS Joly-Amado A Bickford PC Nash KR . CX3CL1/CX3CR1 signaling targets for the treatment of neurodegenerative diseases. Pharmacol Ther. (2022) 231:107989. doi: 10.1016/j.pharmthera.2021.107989. PMID: 34492237

[52] Pawelec P Ziemka-Nalecz M Sypecka J Zalewska T . The impact of the CX3CL1/CX3CR1 axis in neurological disorders. Cells. (2020) 9(10):2277. doi: 10.3390/cells9102277. PMID: 33065974 PMC7600611

[53] Xu H Yang X Xuan X Wu D Zhang J Xu X . STAMBP promotes lung adenocarcinoma metastasis by regulating the EGFR/MAPK signaling pathway. Neoplasia. (2021) 23(6):607–623. doi: 10.1016/j.neo.2021.05.011. PMID: 34102455 PMC8190130

[54] McCullough J Row PE Lorenzo O Doherty M Beynon R Clague MJ . Activation of the endosome-associated ubiquitin isopeptidase AMSH by STAM, a component of the multivesicular body-sorting machinery. Curr Biol. (2006) 16(2):160–165. doi: 10.1016/j.cub.2005.11.073. PMID: 16431367

[55] Ishii N Owada Y Yamada M Miura S Murata K Asao H . Loss of neurons in the hippocampus and cerebral cortex of AMSH-deficient mice. Mol Cell Biol. (2001) 21(24):8626–8637. doi: 10.1128/mcb.21.24.8626-8637.2001. PMID: 11713295 PMC100023

[56] Lin S Xing H Zang T Ruan X Wo L He M . Sirtuins in mitochondrial stress: indispensable helpers behind the scenes. Ageing Res Rev. (2018) 44:22–32. doi: 10.1016/j.arr.2018.03.006. PMID: 29580919

[57] Lin L Guo Z He E Long X Wang D Zhang Y . SIRT2 regulates extracellular vesicle-mediated liver-bone communication. Nat Metab. (2023) 5(5):821–841. doi: 10.1038/s42255-023-00803-0. PMID: 37188819 PMC10229428

[58] Wang Y Yang J Hong T Chen X Cui L . SIRT2: controversy and multiple roles in disease and physiology. Ageing Res Rev. (2019) 55:100961. doi: 10.1016/j.arr.2019.100961. PMID: 31505260

[59] Nagar N Naidu G Panda SK Gulati K Singh RP Poluri KM . Elucidating the role of chemokines in inflammaging associated atherosclerotic cardiovascular diseases. Mech Ageing Dev. (2024) 220:111944. doi: 10.1016/j.mad.2024.111944. PMID: 38782074

[60] Sonawani A Kharche S Dasgupta D Sengupta D . Insights into the dynamic interactions at chemokine-receptor interfaces and mechanistic models of chemokine binding. J Struct Biol. (2022) 214(3):107877. doi: 10.1016/j.jsb.2022.107877. PMID: 35750237

[61] Panezai J Ghaffar A Altamash M Sundqvist KG Engström PE Larsson A . Correlation of serum cytokines, chemokines, growth factors and enzymes with periodontal disease parameters. PLoS One. (2017) 12(11):e0188945. doi: 10.1371/journal.pone.0188945. PMID: 29190740 PMC5708747

[62] Eckersall PD Bell R . Acute phase proteins: biomarkers of infection and inflammation in veterinary medicine. Vet J. (2010) 185:23–27. doi: 10.1016/j.tvjl.2010.04.009. PMID: 20621712

[63] Berg DD Giugliano RP Ruff CT Tang M Im K Jarolim P . A targeted proteomic approach identifies novel biomarkers of arterial thromboembolic risk in ENGAGE AF-TIMI 48. J Am Coll Cardiol. (2021) 78(6):634–636. doi: 10.1016/j.jacc.2021.06.008. PMID: 34353540 PMC8857944

[64] Baysoy A Bai Z Satija R Fan R . The technological landscape and applications of single-cell multi-omics. Nat Rev Mol Cell Biol. (2023) 24(10):695–713. doi: 10.1038/s41580-023-00615-w. PMID: 37280296 PMC10242609

